# Personalized Health Systems—Past, Present, and Future of Research Development and Implementation in Real-Life Environment

**DOI:** 10.3389/fmed.2019.00149

**Published:** 2019-07-31

**Authors:** Hadas Lewy, Refael Barkan, Tomer Sela

**Affiliations:** ^1^Research, Innovation and International Ventures Authority, Holon Institute of Technology, Holon, Israel; ^2^Clalit Healthcare, Research Institute, Tel-Aviv, Israel; ^3^Clalit Healthcare, Online Division, Tel-Aviv, Israel

**Keywords:** personal health systems, real-life pilots, ongoing evaluation, service adaptation, data-innovation

## Abstract

Personal health systems (PHS) are designed to provide the individual with tailored care while enabling the healthcare system to deliver high-quality care to large populations and maintain a sustainable system. Solutions using electronic health records (EHRs) that include predictive models for the risk of disease onset and deterioration enable the care provider to better identify and treat patients with chronic disease and provide personalized prevention. These tools are well-accepted by doctors and have been proven to improve health outcomes and reduce costs. Integrated telecare programs were implemented for comorbid patients showing improved clinical outcomes self-management and quality of life (QoL). However, different patient populations benefit in different ways from these care plans, and thus, continuous evaluation, service adaptation in a real-life environment set with clear outcome measures, is required for best results. The challenge of the PHS today is to acquire patient-generated data (PGD) and behavioral and patient-reported outcomes (PROs) for PHS development that can be combined with existing clinical data. Some initiatives of healthcare organizations [health maintenance organizations (HMOs)] in Israel demonstrate how this goal can be achieved with relatively small efforts by using a stepwise and agile approach to service implementation that improve service by enabling adoption and adaptation of the service in the short term while collecting data for advanced PHS development in the long term. This approach, combined with programs and incentive payments at the national level, creates an environment and infrastructure for collaboration between healthcare, academia, and industry for research, development, and implementation of future PHS. This article presents examples of PHS development and implementation from the Israeli healthcare system. We discuss the lessons learned and suggest new approaches for research, development implementation, and evaluation of PHS that will address the needs of future healthcare.

## Background

Personal health systems (PHS) refer to a wide range of services used to address the needs of the individual patient while enabling the healthcare (HC) system to cope with the challenges of treating large populations providing high-quality care, overcoming disparities, and inequalities in care delivery, and maintaining a sustainable system.

The first generation of digital tools for PHS focused on electronic health record (EHR) data that were used for the development of Decision Support Systems (DSS), implemented online with guidelines for clinicians. Today, predictive models are using real-time data from EHR and management systems to identify patients at risk and provide timely intervention and personalized care. These tools already show improvement in clinical outcomes and cost–benefits (1).

The next generations of PHS using telemedicine were implemented in the last 15 years, for the treatment of comorbid patients and used data from EHRs and the patients. These programs were first implemented as pilots that failed to show clear evidence: clinical and economic. Only in places where the implementation moved beyond pilots to the level of services with ongoing assessment, evaluation, and adaptation is there evidence for clinical and cost–benefits (2).

Today, online services such as pediatrics online, general practitioner (GP) online, and teledermatology are being implemented with a short-term goal to provide better service and high-quality clinical care. However, in the long term, it allows collecting valuable data for further development of PHS. Presently, we are at the beginning of an era of researching and developing analytical tools using big data collected from patients such as behavioral, emotional, and patient-reported outcomes (PROs) and combining them with clinical data (lab, medications, and diagnosis).

The driving forces for the next generation of PHS implementation include the need for service improvement, economic pressures, and HC professional shortages as well as lesson learned from past experience of PHS implementation. The growing need and expectation of patients and other care providers for online, 24/7 personalized services create pressure on health maintenance organizations (HMOs) to implement PHS. Patients are becoming more involved in the design of new services and drive a change in their design development, implementation, and outcome measurements. Shortages of resources (economic and HC professionals) drive HMOs to collaborate with the industry and the academia in the design implementation and evaluation processes (3).

These changes create challenges for old R&D, implementation, and evaluation methodologies. As we move from a world using one-site secured, validated information to “everywhere anytime services” that use personal non-HC devices to produce “non-validated” data, we are challenging existing privacy and security systems.

Presently, we can identify some examples where PHS are already implemented and lessons have been learned. We can also point to initiatives where infrastructures for data sharing are established and create collaborations between academia, HMOs, and industry that develop future PHS for the benefit of the patients, care organizations, and the country at the health population, industry, and economic levels.

This article presents examples of PHS development and implementation from the Israeli HC system in the last 10 years and the actions and infrastructures set for R&D for the future PHS in the HC system. We discuss the lessons learned and suggest new approaches for research, development implementation, and evaluation of PHS that will address the needs of future HC.

## Electronic Health Record as the Basis for Personalized Care

The main challenge in the treatment of large populations is providing personalized care with proactive preventive care, early detection, timely intervention, and disease management. Over the last 10 years, HMOs used their unique asset of data to develop and implement intelligent tools for identifying of patients at risk, prediction of deterioration, and alerts for early intervention.

The Electronic health record adoption started in Israel during the 1990s, and these are widely used by all care providers. Data from EHR, hospitals, and management systems serve as the basis for developing advanced analytical tools for management and treatment. The data are longitudinal and include both claims and clinical data (demographic, diagnoses, labs, imaging, cost, medications, and health services consumption). To overcome security and interoperability barriers, the Israeli Ministry of Health (MOH) established a national system for the exchange of a set of clinical data between hospitals and community care without any direct integration. Quality of care and financial benchmarks are all part of the online system that can demonstrate measurable outcomes. The community care system provides clinical and economic targets for treatment using analytical tools and guidelines for the caregiver. In Maccabi, Health Added Value (HVA) defines health indicators that are translated into specific performance measures integrating quality of care, patient satisfaction, and costs (1, 4). HVA enables measuring performance over time, clinical care outcomes (such as well-controlled diabetic patients), and perceived quality of care directly connected to care cost (5). This approach is combined with other analytical tools for patient classification and stratification, improved clinical outcomes in screening, and chronic disease management. In Clalit, the system is using calculated scores indicating the urgency for intervention with DSS for the treatment of patients in the community [diabetes, hypertension, congestive heart failure (CHF), and low-density lipoprotein (LDL)].

In addition, HMOs are implementing predictive models that identify patients at high risk (HR) and are focusing on intervention. The effectiveness of these models has been evaluated over a long period on large populations and already shows success. For example, a personalized proactive care plan based on a predictive model identifying individuals at HR for renal failure (8% of the patients with highest risk for end stage) over a 5 year period was implemented in 2012. This improved outcomes while reducing cost. Another predictive model for colon cancer based on a routine blood test was implemented 4 years ago and identifies individuals who would most benefit from colorectal cancer (CRC) screening (6). A care program based on a 30 day readmission prediction model was implemented in 2012 resulted in a reduction of 12.3% in readmission among HR patients (7, 8). These programs are being applied to the overall population, combined EHR data, artificial intelligence (AI) tools, and care programs implemented as part of the routine service. They improve clinical outcomes and provide cost–benefits. Furthermore, the results are presented to managers and doctors to create transparency and open discussion about ongoing improvement of targets and quality measures while increasing doctors' acceptance (9). The success of these programs suggests that care programs based on big data designed for the prevention and management of large populations should be implemented as services and evaluated for long periods in a real-life environment and not as pilots.

## Integrated Care and Personal Health Systems

Chronic disease prevalence is increasing, making up 60% of the global disease burden. Comorbid patients have higher in-hospital stay and mortality rates, higher average hospital costs, low quality of life (QoL), and medication morbidity (10). Therefore, a special focus was put on comorbid treatment including care coordination, patient education, and self-care. Pilots that were performed in different countries on different cohorts with different care programs included limited numbers of patients for relatively short periods and failed to show clear cost-effectiveness and clinical outcomes. As a result of these pilots, there was an agreement among care providers that telecare should be implemented as part of the health service. In Israel, PHS programs were developed in both large HMOs, and both programs provided care coordination by nurses supporting the physicians. The CC-MAP program started in 2010. A personalized care plan is designed by a nurse and a doctor, and the patient receives education for self-treatment. The system uses patient scores for prioritization of interventions and guidelines for treatment. Learning from the first generation of pilots, this program was designed and implemented as a service. The first step of the implementation includes a pilot with 1,000 patients to examine the feasibility and for adjustment of the service before development and integration. There is ongoing evaluation and service adaptation, including adjustment of inclusion criteria (11).

Maccabi established in 2012 a nurse telecare center that provides the GP support for managing comorbid patients. The aim is to preserve/enhance patient physical and mental QoL, empower, and improve compliance rates, for improved health outcomes. The service was first launched as pilot for CHF patients including daily home monitoring (weight, heart rate, and blood pressure). However, the actual added value of the monitoring was not clear. The service proved improvement in QoL, mental condition, and health behavior (12). These results along with the shortage in HC professionals led to the decision to establish a telecare center. The service, designed for 10,000 patients with diverse conditions, such as fragile, diabetic, stoma CHF, and chronic obstructive pulmonary disease (COPD) patients, was launched without sensors. Patients were asked to self-monitor as needed, enabling personalization according to need and personal abilities (tablets, internet/mobile, and self-monitoring).

The outcomes differed between patient groups. Diabetic patients showed a significant reduction in HbA1c levels within 6 months. Seventy-seven percent of the patients were discharged from the program after reaching treatment goals. HbA1C levels remained stable, indicating that training and patient engagement produce long-term outcomes. In frail patients, the 389 frail patients treated during 2015 showed a 30% reduction in hospitalization days and lower overall average monthly costs compared to control (2). All patient groups showed a significant improvement in health behavior (vaccination, physical activity, and healthy eating), QoL, and mental health. Thus, the service is cost-effective and improves health outcomes in specific patient populations, indicating that there is a need for long periods for evaluation and continuous adaptation to yield best outcomes. Each patient population benefits in a different way from the service, and the evidence base of the actual benefits for each patient population cannot be obtained in a pilot applying the care model to a specific population for a limited period. Therefore, the care program should use “real-life pilots” and be adopted for a long time by each population, with relevant and realistic targets outcome measures accompanied by ongoing research and evaluation assessing each parameter such as monitoring devices at the level of a specific population.

## Online Service and Data Collection for Personal Health Systems

Although analytical tools as described above help in delivering high-quality care for large populations, efficient PHS requires patient involvement in the treatment process. This approach includes data sharing and transparency, patient education, involvement, and the use of patient-generated data (PGD) as part of the clinical care. This treatment approach was successfully implemented in integrated care programs as described above. However, these programs, designed for comorbid patients, are expensive. They showed different benefits for different patient populations; thus, they can be delivered to relatively small populations. These conclusions led to the development of online tools for data sharing, education, and self-management.

The first tools developed for data sharing focused mainly on presenting clinical information to the patient (lab results and medication list), combined with alerts and reminders for follow-up for both patients and doctors. To overcome security barriers, this was done by exporting a partial data set from the EHR to an external domain without enabling patient access to their EHR. Personal health records (PHRs), which combine data from EHR and PGD, were implemented to help patients become active participants in their own care. In order to overcome legal, data validation, security, and interoperability barriers, the uploaded PGD is not integrated into the EHR and is ignored in the care process. In parallel to the development of HC systems available through the HMOs, online tools became available in the public domain. Patients are using social media, medical forums, doctors' online advice, well-being apps, and smart watches for self-monitoring. In practice, doctors are already interacting with patients *via* existing non-HC channels such as text/whatsApp/phone. These data are not secured, collected, or used for development of the next generation of AI for PHS. As new services develop for homecare, the opportunity for data collection drives the HMOs to integrate these services and collect the data for the development of AI tools for diagnosis, early detection, and DSS for treatment. Two approaches exist for the implementation of such tools by HMOs: the first approach is a stepwise approach in which the service may not be fully integrated at the first phase but will enable quick adoption and user education while performing evaluation and adaptation to the real-life environment until reaching full implementation. The second approach is to implement only fully integrated services.

Examples for stepwise approach can be demonstrated in the *Pregnancy follow-up* service launched in 2012 and first implemented as a non-integrated application based on patient-entered clinical data. It includes a user forum and information created by HMO clinicians. To avoid data security issues, the application directs Clalit patients to the organizational site, and from there, all actions are enabled for Clalit patients. Non-Clalit members can use the app without the organizational links. The app became very popular, and the activity online has reached 500,000 entries/month by 35,000 users/month. The online activity provided information regarding the main interest of the users that was analyzed last year and provided ideas for further developments. These were validated with users in focus groups. As a result, starting from 2019, this app will be integrated into EHR and will include a personal avatar-like advisor that accompanies them through their pregnancy, providing an innovative personal user experience. All data will be secured according to privacy and security regulation and will be used for future developments.

This stepwise approach was also used in *GP virtual visits* launched in July 2018. Patients purchase a sensor kit (statoscope, camera device for ears and throat, and blood pressure) and schedule an appointment through an app. The patient self-measures and the data are saved to the cloud using an anonymous ID. This is presented on a doctors' screen during the remote visit. The data are not yet integrated, and the doctor documents the visit in the traditional way on the EHR, performing the virtual visit in the same way as a face-to-face visit with the same duration of 6 min on average. Within the first 5 months, 3,000 kits were purchased. The usability rate is many hundreds per week, and the average users' age is 48 years ranging from families with children (56%) to age 95. Seventy-five percent of chronic patients (compared to 60% of the overall population) are using the service, indicating a high percentage of users among this group. No gender difference was found. The service provided to patients at home 24/7 shortens scheduling time from 2 days to 1 h. While patients and doctors adopt the new service, valuable data are collected. These data will serve as the database for validation of home monitoring data and R&D of AI tools for primary care. The information coming from the GP documentation compared to the sensors data collected on the cloud, on a large population in real life, will enable research of diagnosis and DSS for treatment. This may help to provide high-quality care using AI in primary care as exists in imaging in which AI tools are validated and implemented (13).

*Dermatology online* was launched 3 years ago despite doctors' doubts about using a photo for diagnosis. However, as they use the system, they trust it and adopt it. This service shortens scheduling time from 2 weeks to 1 day. Today the service provides 2,500 consultations/month and is continuously growing. A total of 80,000 images from personal smart phones already sent for diagnosis are now also being used for R&D of AI tools for skin conditions in collaboration with start-up companies. This service changes the treatment approach by using photos from “non-HC” devices with minimal security sent to an HC organization and used for care. Its success opens opportunities for data collection and integration in other domains. For example, *PROs* are becoming major indicator for treatment success. Sheba hospital has implemented PROs collected for various conditions since 2016 as part of the follow-up process. Data are presented on a doctor's screen with individual target outcomes and in parallel are being used to develop AI for prediction for personalized outcomes (14).

The *child development online* service was designed to enable prioritization of care by EHR pre-screening when an appointment is scheduled. This approach was found to be non-effective and time-consuming as the information in the EHR is not complete. Only 10% of the patients could benefit from it. As a result, the service was redesigned, and all patients can use an online service without prioritization. This experience emphasizes the importance of an agile methodology and a non-integrated service in the first stage of implementation.

A fully integrated service approach was implemented in the *home urinary test* for pregnant women using a home kit and a smartphone app, launched in May 2018. The test is sent through a dedicated app to the cloud, validated, archived, and presented to the care provider on the EHR. During the first 6 months, 900 tests (400 patients) were performed (15). This approach enables the doctor to have the results in the EHR. However, it has no advantage in development of innovative analytical tools for care.

These examples demonstrate the use of an agile methodology in service development and implementation to create value in the short term by improving service availability, user experience, and satisfaction and in the long term by establishing an infrastructure for data collection and R&D for advanced PHS. The gradual process of implementation without full integration in the first phase also enables adaptation and changes in service design until reaching an optimal service model. Furthermore, this approach of research, development, and validation of the PHS are based on real-life data and the target population. Once the tool is validated, the integration process will be seamless since the service is already implemented.

## Conclusions and Future for Personal Health Systems

Personal health systems were developed as part of EHR and HC management system development. The existing infrastructure provides a valuable asset of big data used for the development of AI tools for population management in health and disease. These tools improve clinical outcomes and reduce costs. Today, community care systems use online AI tools that combine clinical outcomes with economic and patient satisfaction outcome measurements for care. As the treatment approach moved from reactive to proactive care and includes prevention, early detection, and timely intervention, there is a need to involve the patient in the care process and complement the clinical data produced at the point of care with PGD that includes home monitoring, behavior, and PROs. Pilots designed for specific populations, usually complex patients, for a limited time failed to demonstrate improved clinical outcomes and cost-effectiveness. It was shown that not all subpopulations benefit from each specific service in the same way, and it takes several years to demonstrate improvement in clinical outcomes and cost–benefits in different patient populations. Therefore, it is recommended to move into “living pilots” with clear outcome measures implemented in a real-life environment while performing ongoing evaluation and adaptation of the service using an agile methodology.

Personal health systems development requires real-life data from large populations. Creating big data sets for personalized care from various populations requires investment, time, and involvement of HMOs and the regulator. The data that exist today in HMOs are not available to the research community and industry, limiting the development potential of innovative PHS. In HMOs, there are no programs designed to collect data from healthy people, limiting our ability to develop PHS for the overall population. On the other hand, data coming from citizens using wearables connected to commercial services cannot be used by HMOs for regulatory reasons related to the health data protection law and also to general privacy law. Presently, the most available data originate from the EHR with only small data sets of PGD. Furthermore, there is not enough knowledge on how to use PGD for diagnosis, prediction for disease onset, deterioration, and stratification. However, we do have data that can be used for the development of PHS.

Health maintenance organizations implement online services that in the short term improve quality of service and user satisfaction (both doctors and patients) but in the long term serve to build infrastructure for data collection of real-life data that will be used for R&D of advanced PHS to improve quality of care with minimal additional cost. Furthermore, by using this approach, we enable the users to adopt the system and overcome cultural and organizational barriers. Organizations implement solutions that use personal devices: smart phones and home monitoring (not just medical devices). They integrate data that were created outside the point of care, patient “non-validated” data, and subjective data such as PROs and use cloud computing for data storage and security. This changes old concepts and brings new challenges, driving HMOs to collaborate with the industry and the academic community for research and development of advanced solutions and new models of care.

To facilitate collaboration between all stakeholders, the Israeli government has launched several national programs that will support research, development, and implementation of advanced PHS for the benefit of the population and the industry. Funding is offered to HMOs to develop platforms that will enable access to anonymized, updated HC data for industry and researchers. In addition, a special program was established for genetic data that will be available to researchers in addition to the complementing clinical data coming from HMOs. In parallel, the MOH is funding a pilot program to encourage implementation of new technologies at early stages. This will enable the industry and HC system to collaborate and implement innovative technologies.

The systems developed in HMOs in Israel created an effective infrastructure for development and use of PHS. However, when moving to the next phase in which data from patients and HC will be collected, combined, analyzed, and used for HC, there is a need for collaboration between the academic, industry, and HC sectors. The present policy and incentive payments invested in this domain, together with the ongoing activity of HMOs for the improvement of service while creating infrastructure for R&D, will produce outcomes in the coming years.

## Author Contributions

All authors listed have made substantial, direct and intellectual contribution to the work, and approved it for publication.

### Conflict of Interest Statement

The authors declare that the research was conducted in the absence of any commercial or financial relationships that could be construed as a potential conflict of interest.
